# Herbicidal Control Potential of the Endophytic Bacterium *B. pseudorignonensis* BFYBC-8 Isolated from *E. crus-galli* Seeds

**DOI:** 10.3390/microorganisms13102293

**Published:** 2025-10-02

**Authors:** Dashan Yang, Quanlong He, Qingling Wang, Jing Zhou, Haiyan Ke, Xin Wen, Jiawei Pan, Yi Zhou, Jianwei Jiang

**Affiliations:** College of Agriculture, Yangtze University, Jingzhou 434025, China; dysonyuong1216@126.com (D.Y.); 13657161586@163.com (Q.H.); 18695006691@163.com (Q.W.); zjdwwwswsww@163.com (J.Z.); 151711118719@163.com (H.K.); 13341571695@139.com (X.W.); 15216838628@163.com (J.P.)

**Keywords:** plant-microbe interaction, seed endophytic bacteria, drought stress tolerance, plant growth promotion, barnyard grass

## Abstract

The long-term application of traditional chemical herbicides has caused a significant escalation in herbicide resistance of barnyard grass (*Echinochloa crus-galli*). As an eco-friendly alternative, biological herbicides demonstrate substantial application potential. Acknowledging the growing herbicide resistance of *E. crus-galli*, this study aimed to screen target bacteria with inhibitory effects on the growth for bio-herbicide development. By using ungerminated *E. crus-galli* seeds as the screening substrate, a bacterial strain (BFYBC-8) with potent inhibitory activity was isolated and identified as *Brucella pseudorignonensis*. Pot experiments revealed that inoculation with *B. pseudorignonensis* BFYBC-8 significantly suppressed *E. crus-galli* growth, reducing plant height by 16.7% and root length by 85.1%, while markedly inhibiting biomass accumulation. Fluorescent labeling with green fluorescent protein (GFP) showed that BFYBC-8 successfully colonized the root intercellular spaces of *E. crus-galli* and extended continuously along the tissue matrix. Additionally, the strain’s supernatant metabolic products exhibited exceptional thermostability: inhibitory activity against *E. crus-galli* was maintained after thermal treatment at 28 °C, 60 °C, 80 °C, and 100 °C. Crucially, the bacterium displayed no toxicity to agronomically important crops such as rice, wheat, and corn. This study highlights *B. pseudorignonensis* BFYBC-8 as a promising candidate for bioherbicide development and provides an important reference for applying seed-associated pathogenic bacteria in developing bioherbicides for sustainable weed management.

## 1. Introduction

*Echinochloa crus-galli*, an aggressive annual grass weed, poses a significant threat to crop yields, particularly in rice and wheat fields, and is classified as a major noxious weed [1]. Current management strategies rely heavily on conventional chemical herbicides; however, prolonged use of these agents has diminished the efficacy of previously effective herbicides targeting this weed in recent years [2]. The resilience and adaptability of *E. crus-galli*, including its documented resistance to various modes of action, further hinder the effectiveness of traditional agricultural practices and chemical control measures [3]. These challenges underscore the urgent need for sustainable, eco-friendly herbicides that are less prone to resistance development [4].

Microbial-derived herbicides, originating from plant pathogenic microorganisms or their metabolites, represent a promising alternative for weed management [5]. As an environmentally friendly weed management technology, microbial-derived herbicides exhibit remarkable diversity and complexity in their modes of action and taxonomic categories. Based on differences in mechanisms and active ingredients, microbial-derived herbicides can be systematically categorized into three major classes. (1) Living Microbial Herbicides: These agents utilize fungi, bacteria, or viruses to directly infect weed tissues, colonize sensitive plant structures, and disrupt normal physiological processes through nutrient competition and toxin secretion, e.g., cell wall-degrading enzymes and secondary metabolites [6,7]. (2) Allelopathic Microorganisms: These microorganisms release allelochemicals such as phenolic acids, terpenoids, and alkaloids that interfere with weed seed germination, root development, and photosynthesis [8]. (3) Symbiotic Microorganisms: These agents inhibit weeds through nutrient competition or by inducing systemic resistance in host plants [9].

These biocontrol agents are abundant and can be isolated from diverse microbial communities [10]. Recent studies have identified over 80 microorganisms across 40 genera with weed-suppressing properties, notably including nine genera demonstrating exceptional weed control efficacy: *Alternaria, Ascochyta*, *Cercospora*, *Colletotrichum*, *Entyloma*, *Fusarium*, *Phytophthora*, *Puccinia*, and *Sclerotinia* [11]. Research findings indicate that specific pathogenic strains targeting *E. crus-galli*, *Eleusine indica*, *Leptochloa chinensis*, and *Digitaria sanguinalis* effectively suppress weed growth without harming crops such as cotton, wheat, and rice [12]. Besides fungi, bacterial pathogens also primarily affect weed growth by producing toxins, which mainly include Gram-positive and Gram-negative bacteria [6]. Studies have shown that *Pseudomonas fluorescens* has a certain control effect on *Bromus tectorum* [13]; *Xanthomonas campestris* can inhibit the growth of *Erigeron canadensis* [14]; and pathogenic strains of *Enterobacter* spp. are effective against both *E. crus-galli* and *Portulaca oleracea* [15]. Despite this potential, bioherbicide products based on these strains have not yet achieved commercial viability [16].

The 1992 World Conference on Environment and Development set a goal for biopesticides to account for 60% of total pesticide production by 2000, a target that remains unmet. The shift toward biopesticides, particularly biological herbicides as alternatives to chemical herbicides, is a global priority [17,18]. The annual global consumption of herbicides in 2020 was 1.397 million metric tons [19,20]. The world’s leading consumers of pesticides were China (14.0 kg·ha^−1^), Japan (11.0 kg·ha^−1^), and the United States (4.5 kg·ha^−1^), while the global average stood at 3.0 kg·ha^−1^ [21]. In China, where over 40,000 pesticide products are registered, biological pesticides account for only 4.78% of the total, with virtually no commercially successful bioherbicide varieties available [17]. This disparity underscores the urgent need for advancing research and development in microbial-derived herbicides [22].

This study aims to select a plant-pathogenic bacterial candidate from non-germinated *E. crus-galli* seeds, to identify the bacterium based on cultural, molecular, and biochemical characterization, and to evaluate and analyze its herbicidal potential against *E. crus-galli.* It may provide an important reference for applying seed-associated pathogenic bacteria in developing bioherbicides for sustainable weed management.

## 2. Materials and Methods

### 2.1. Plant Material

In July 2023, seeds of *E. crus-galli* were collected from arid farmland in Mapaoquan Village (39°91′ N, 116°41′ E), Jingzhou City, Hubei Province, China. The *Echinochloa crus-galli* in this region exhibited stunted growth, appearing short and yellow. Poorly growing plants were selected for seed collection. The seeds were stored in clean kraft paper bags and kept at 4 °C for subsequent use. A total of 500 g of *E. crus-galli* seeds was selected for soaking and germination, while non-germinating seeds were reserved for future isolation. Seeds of rice (*Oryza sativa* L. cv. Chuangliangyou 669), wheat (*Triticum aestivum* L. cv. Xinong 979), and maize (*Zea mays* L. cv. Zhengdan 985) were procured from commercial seed suppliers.

### 2.2. Isolation and Screening of Pathogenic Bacteria from E. crus-galli Seeds

*E. crus-galli* seeds were surface-disinfected. The specific disinfection steps were as follows: the seeds were immersed in 5% sodium hypochlorite solution for 2 min, and then transferred to 75% alcohol solution for 30 s to further eliminate the microorganisms on the surface of the seeds. The success of this procedure was assessed by plating 100 µL of the final sterile rinse water onto Luria–Bertani (LA) agar medium to check for any contaminating microbes. Then the surface disinfected seeds were ground into powder, and diluted to concentrations of 10^−4^ and 10^−5^ using sterile water. Aliquots of 100 μL from each dilution were spread onto Luria–Bertani (LB) medium (tryptone 10 g/L, NaCl 10 g/L, yeast extract 5 g/L, pH 6.8–7.0), which was then inverted and incubated at 28 °C for 3 days. Bacterial colonies were selected and purified using streak culturing. The purified strains were stored in glycerol suspensions (20%, *v*/*v*) at −80 °C.

For pathogenic strain screening, the isolated bacteria were cultured in 50 mL of LB medium on a rotary shaker at 150 rpm for 48 h at 28 °C. The cultures were centrifuged at 8000 rpm for 10 min, and the bacterial pellets were resuspended in sterile water. The bacterial concentration was adjusted to an optical density (OD_600_) of approximately 1.0 with a spectrophotometer. Surface-disinfected *E. crus-galli* seeds were soaked in the bacterial inoculum for 1 h and then placed on moist filter paper in Petri dishes, with 15 seeds per dish. The control seeds were mock-inoculated with sterile water. The seeds were incubated at 28 °C for 7 days, after which root length and plant height were measured. The pathogenicity of each strain was initially quantified by calculating the inhibition rate of root length and plant height compared to the control.

Four strains (BFYBC-8, BFYBC-11, BFYBC-12, BFYBC-17) exhibited superior pathogenic effects on *E. crus-galli* seeds. In order to ensure the stability of the pathogenic effect of the strain, the established scheme was used to repeat the test again, and BFYBC-8 was determined to be the most pathogenic and stable strain, and it was selected for further research.

### 2.3. Strain Identification

Morphological identification of the pathogenic strain BFYBC-8 was performed by streaking cultures on LB plates. The strain BFYBC-8 was incubated at 28 °C for 24 h to evaluate colony morphology, size, and moisture content. A single colony of strain BFYBC-8 was inoculated, subjected to smearing, fixation, and Gram staining, and examined under a Nikon ECLIPSE Ni-U microscope system (Nikon, Tokyo, Japan) to determine bacterial morphology.

For molecular identification, strain BFYBC-8 were inoculated to LB broth and cultivated at 28 °C with shaking at 200 rpm for 24 h. The genomic DNA was extracted using the FastPure Bacteria DNA Isolation Mini Kit (Vazyme, Nanjing, China). PCR amplification of 16Sr RNA gene region was conducted using universal primers 27F (5′-AGAGTTTGATCCTGGCTCAG-3′) and 1492R (5′-GGTTACCCTTGTACGACTT-3′) [22]. The total PCR reaction was 50 μL including 2.5 μL of DNA template, 25 μL of 2 × Taq PCR super mix, 1 μL of each primer, and 20.5 μL of ddH_2_O. The amplification process had thermal cycling conditions of 94 °C for 3 min, 34 cycles of denaturation at 94 °C for 30 s, annealing temperature at 52 °C for 30 s, and synthesis process at 72 °C for 30 s, an extension of 72 °C for 5 min, and the infinity cooling temperature of 16 °C. The PCR products were purified and sequenced by Qingke Biotechnology Co., Ltd. (BeiJing, China). The obtained sequences were deposited in GenBank database. The relevant sequences were retrieved from GenBank database for the sequence analysis. All the sequences were aligned by using MAFFT v7.525 [23], and edited in MEGA v.7.0 software [24]. A phylogenetic tree was then constructed using MrBayes 3.2.7a software [25], and visualized with Figtree 1.4.4 (http://tree.bio.ed.ac.uk/software/figtree (accessed on 30 May 2025).

### 2.4. Pot Experiment of Pathogenic Strains

A controlled experiment was conducted to assess the pathogenicity of the bacterial strain BFYBC-8. The bacterium were cultured in LB medium at 37 °C and shaker (180 rpm) for 48 h to OD_600_ = 1.0. The culture was then centrifuged at 8000× *g* for 15 min at 4 °C, and the supernatant was filtered through a 0.22 μm sterile filter to obtain the cleared culture supernatant. And a 2:1 (*v*/*v*) mixture of red soil and vermiculite was autoclaved at 121 °C and 101 kPa for 2 h to obtain pathogen-free soil. *E. crus-galli* seeds were selected for uniform size and integrity, surface-disinfected, and then germinated until radicle emergence. The germinated seeds were then transplanted into plastic pots containing an appropriate amount of 500 g sterile soil at 30 seeds per pot, with three biological replicates per treatment. The plants were grown in a greenhouse with the routine light period (12 h light/dark period) at room temperature for 14 days. On the 1st and 3rd day of the experiment, 20 mL supernatant was inoculated, and the control group was inoculated with the same amount of sterile water. 14 days after planting, *E. crus-galli* growth was evaluated by measuring root length, plant height, fresh weight and dry weight (85 °C for 48 h to constant weight).

### 2.5. GFP Labeling and Colonization Test of Pathogenic Strains BFYBC-8

The green fluorescent protein (GFP)-labeled strain BFYBC-8 was developed through conjugation-mediated transformation utilizing the plac-EGFP-Chl-signal-Hyg plasmid system described in Liang et al. [26]. The surface-sterilized *Echinochloa crusgalli* seeds were incubated in a bacterial suspension for 1 h, and then cultured on a moist filter paper in a sterile Petri dish at 28 °C for 7 days. After cultivation, root systems were harvested, rinsed with sterile water, and vertically sectioned using a Leica CM1860UV cryostat microtome (Leica, Wetzlar, Germany). Fluorescent visualization was performed with a Leica DMi8 confocal laser scanning microscope (Leica, Wetzlar, Germany), employing excitation and emission wavelengths of 490 nm and 510 nm, respectively, for GFP detection.

### 2.6. Crop Safety Test of Strain BFYBC-8

The seeds of wheat, rice and corn were sterilized and cultured in sterile soil until the two-leaf stage, during which they were irrigated with sterile water. The strain BFYBC-8 was cultured in LB liquid at 37 °C and shaker (180 rpm) for 48 h until OD_600_ = 1.0. The bacterial suspension obtained from the supernatant was inoculated on the two-leaf stage plants of rice, wheat and corn, 20 mL per pot. An equal volume of sterile water was used as the control. The toxicity of BFYBC-8 to plants was evaluated 14 days after treatment.

The phytotoxic effects of bacterial strain BFYBC-8 on rice, wheat, maize, and *E. crus-galli* were evaluated based on a defined disease severity index. The symptoms on each plant were scored 14 days after treatment using the following rating scale:

Grade 0: No symptoms; plant growth is healthy and normal.

Grade 1: Very mild symptoms; 1–2 leaves show slight yellowing or wilting, but overall plant growth is not significantly affected.

Grade 2: Moderate symptoms; multiple leaves show clear yellowing, wilting, or slight stunting.

Grade 3: Severe symptoms; severe wilting, leaf necrosis, pronounced stunting, or plant death.

Disease incidence (DI) was calculated as the percentage of plants that showed any disease symptoms (plants with a severity grade ≥ 1) among the total number of plants treated in each group.

The formula for disease incidence is: DI (%) = [Number of symptomatic plants/Total number of plants assessed] × 100%.

### 2.7. The Inhibitory Activity and Thermal Stability of Secondary Metabolites of Strain BFYBC-8

Pathogenic strains were cultured in LB broth at 28 °C for 72 h under agitation. The culture was centrifuged at 8000× *g* for 20 min at 4 °C to separate pellet bacterial cells. The supernatant was carefully transferred and filtered through a 0.22 μm sterile membrane filter to ensure sterility. The sterile filtrate was subjected to thermal treatment at 28 °C, 60 °C, 80 °C, and 100 °C for 30 min each. Inoculation experiments were conducted in Petri dishes using *E. crus-galli* seeds. After 7 days of incubation, inhibition rates of root length and plant height were calculated as the percentage reduction compared to the control group.

### 2.8. Component Identification of Strain BFYBC-8 Extract by Liquid Chromatography-Mass Spectrometry (LC-MS)

The strain BFYBC-8 was cultured in LB broth at 28 °C for 72 h under agitation. The culture was centrifuged at 8000× *g* for 20 min at 4 °C to separate pellet bacterial cells. The supernatant was carefully transferred and filtered through a 0.22 μm sterile membrane filter to ensure sterility. Finally, the filtrates were freeze-dried into powder, and dissolved in methanol, diluted (10×, 100×), and filtered before injection. The chromatographic conditions were as follows: Kinetex^®^ F5 column (100 mm × 2.1 mm, 2.6 μm), mobile phase of water (A) and acetonitrile (B) with gradient elution (0–25 min: 5% to 95% B; reset to 5% B at 25.01 min), flow rate of 300 μL/min, and injection volume of 10 μL. Mass spectrometry conditions: ESI^+^/^−^ ionization modes, spray voltage of 5000 V, ion source temperature of 500 °C, and mass scan range of *m*/*z* 100–1250. Data processing was performed using SCIEX OS 1.7.0 and main components were identified by comparison with the database.

### 2.9. Experimental Apparatus

Details of all instruments involved in the process of the test are shown in the Table 1.

### 2.10. Data Analysis

One-way ANOVA was used to analyze the experimental data using SPSS 17.0 software (SPSS Inc., Chicago, IL, USA). Prior to analysis, the percentage data (inhibition rates) were subjected to arcsine square root transformation to stabilize variance and better meet the assumptions of ANOVA. The mean values of the various treatment groups were compared using Duncan’s multiple range test, which provides a balanced approach to controlling Type I error while maintaining statistical power for multiple comparisons. A significance level of *p* ≤ 0.05 was used to establish statistical significance.

Compute the root length inhibition rate using Formula (1) and the plant height inhibition rate using Formula (2).

Root length inhibition rate (%) = ((control root length − treated root length)/control root length) × 100(1)

Plant height inhibition rate (%) = (control plant height − treated plant height)/control plant height × 100(2)

## 3. Results

### 3.1. Isolation and Screening of Pathogenic Strains

Twenty-eight bacterial strains were isolated and purified from *Echinochloa crus-galli* seeds, which were designated BFYBC-1 to BFYBC-28. Pathogenicity screening identified four highly pathogenic strains from the 28 isolates. Although all four strains inhibited plant growth (25.1% for BFYBC-8, 25.7% for BFYBC-11, 30.3% for BFYBC-12, and 24.4% for BFYBC-17), only BFYBC-8 exhibited strong suppression of root elongation, with an inhibition rate of 86.3% (Table 2).

**Figure 1:**
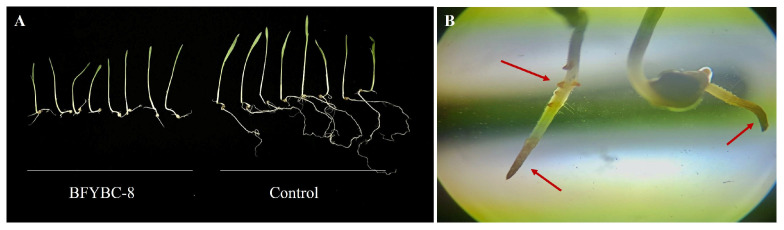
Pathogenicity test of *B. pseudorignonensis* BFYBC-8 on *barnyard grass*. (**A**) Comparison of cumulative biomass between bacterial supernatant treatment and control (water treatment). Electron microscope photographs of *Echinochloa crusgalli* roots infected by strain BFYBC-8 (**B**). The area referred to by the arrow is the part that may be infected by bacteria.

As shown in Table 3, in the *E. crus-galli* experiment, BFYBC-8 exhibited inhibition rates of 16.7% on plant height and 85.1% on root length. In contrast, BFYBC-11, BFYBC-12, and BFYBC-17 demonstrated significantly weaker inhibitory effects on plant height, with inhibition rates of only 1.7%, 1.4%, and 2.8%, respectively. BFYBC-8 showed significantly higher inhibition rates compared to the other strains (*p* < 0.05). Although BFYBC-11, BFYBC-12, and BFYBC-17 initially showed some inhibitory effect on plant height, they were unstable in subsequent tests and thus were not further investigated. BFYBC-8 exhibited strong stability and potent inhibitory effect on root length. As shown in Table 3 and Figure 1A, this strain significantly suppressed both plant height and root elongation. Based on its efficacy and stability, BFYBC-8 was selected as a promising bioherbicide candidate against barnyard grass. Microscopic analysis revealed (Figure 1B) that BFYBC-8 inhibited the growth of barnyard grass by targeting root tip and secondary root development. Some root hairs turned yellow and the roots were significantly shortened.

### 3.2. Identification of Strain BFYBC-8

**Figure 2:**
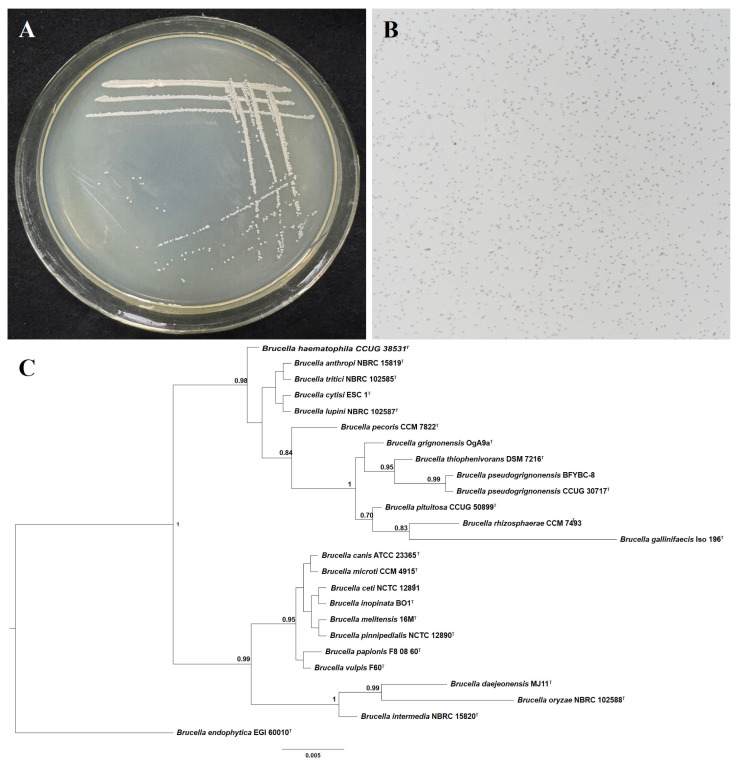
Identification of *B. endophytica* BFYBC-8. (**A**) Colony morphology. (**B**) Gram staining. (**C**) Bayesian tree of *B. endophytica* BFYBC-8 inferred from 16S rRNA gene sequences. Bayesian posterior probabilities > 0.5 are given at the nodes. *Brucella endophytica* strain EGI 6000 was selected as the outgroup. ^T^: type strain. Scale bar: (**B**) = 5 µm.

Strain BFYBC-8 was cultured on LB plates at 28 °C for 24 h exhibiting characteristic colonial morphology: milky-white pigmentation, opaque surface, circular morphology (Figure 2A). Prolonged incubation (5–7 days) the colonies underwent a color transition, becoming paler and more transparent. Gram staining followed by oil immersion microscopy revealed intracellular red pigmentation in the cells, confirming Gram-negative classification (Figure 2B). The 16S rDNA sequence of strain BFYBC-8 (accession no. PQ460246) exhibited 100% similarity to several reference sequences within the genus *Brucella*, including that of *B. endophytica*. In the Bayesian inference phylogenetic tree constructed based on 16S rDNA gene sequences, strain BFYBC-8 formed a stable cluster with the type strain *B. pseudorignonensis* CCUG 30717 (Figure 2C). However, given that the 16S rDNA gene lacks the resolution for definitive species-level discrimination within *Brucella*, our results robustly support the assignment of strain BFYBC-8 to the genus *Brucella*, and we conservatively designate it as *B. pseudorignonensis* BFYBC-8.

### 3.3. Pot Experiment of Pathogenic Strains B. pseudorignonensis BFYBC-8

*Brucella pseudorignonensis* BFYBC-8 exhibited significant inhibitory effects on both *E. crus-galli* seed germination and seedling growth. In seed germination assays, treatment with *B. pseudorignonensis* BFYBC-8 secondary metabolites (200 μg/mL) reduced the germination rate to 59.9%, a 15.1% decrease compared to the sterile water control (70.5%). The germination process was delayed, with the cumulative germination rate on day 3 being only 43.6% relative the control.

**Figure 3:**
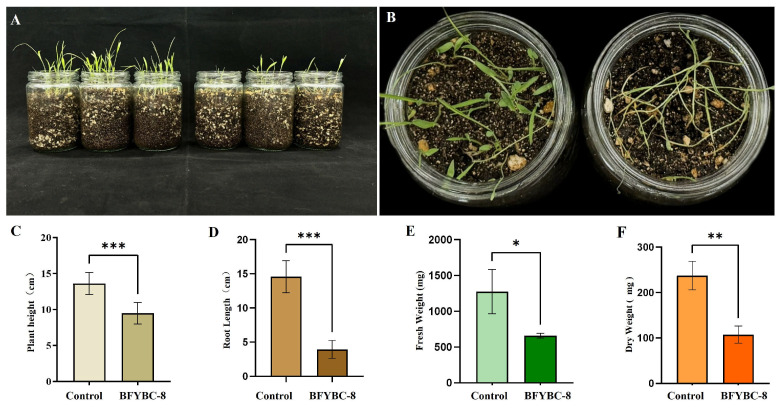
The growth appearance (**A**,**B**), plant height (**C**), root length (**D**), fresh weight (**E**), dry weight (**F**) of *E. crus-galli* inoculated with *B. pseudorignonensis* BFYBC-8 under pot culture conditions at 14 day. *: 0.01 < *p* < 0.05; **: 0.001 < *p* < 0.01; ***: *p* < 0.001.

Pot experiments further confirmed the strong pathogenicity of strain BFYBC-8 (Figure 3). *E. crus-galli* treated with *B. pseudorignonensis* BFYBC-8 showed a 30.1% reduction in root length (9.5 ± 0.3 cm vs. 13.6 ± 0.2 cm) and a 73.3% reduction in plant height (3.9 ± 0.1 cm vs. 14.6 ± 0.3 cm) compared to the control. Fresh weight (0.64 ± 0.05 g vs. 1.27 ± 0.31 g) and dry weight (107.3 ± 18.7 mg vs. 237.3 ± 31.6 mg) were reduced by 49.6% and 54.8%, respectively *(p* < 0.001) (Figure 3C–F). The comprehensive morphological evaluation recorded the characteristic symptoms of the plants inoculated with the pathogen. Compared with the control group, the plants inoculated with BFYBC-8 were short, the stems and leaves were thin, and there was a significant lodging phenomenon (Figure 3A,B). These results indicate that *B. pseudorignonensis* BFYBC-8 inhibits seed germination via secondary metabolites and suppresses seedling morphogenesis and biomass accumulation through direct infection.

### 3.4. Colonization of B. pseudorignonensisBFYBC-8

**Figure 4:**
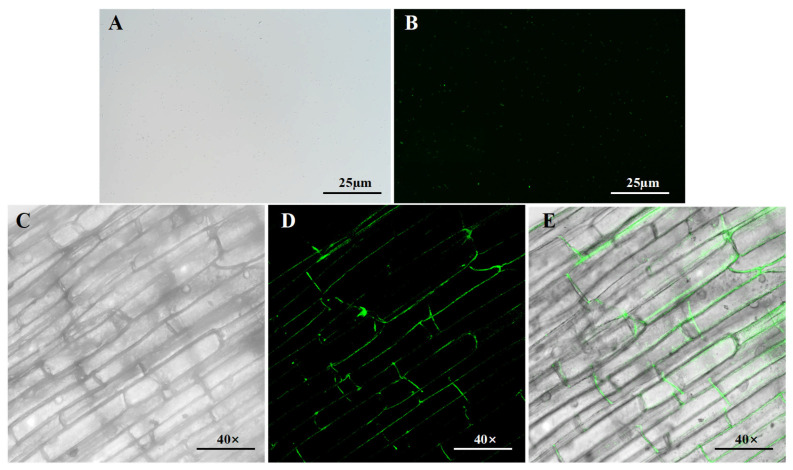
Confocal laser scanning microscopy images revealed the localization of GFP-labeled *B. pseudorignonensis* BFYBC-8 in *E. crus-galli* roots. (**A**) Bright-field and (**B**) fluorescence imaging of GFP-labeled BFYBC-8. (**C**–**E**) High-magnification views of root-associated bacteria *B. pseudorignonensis* BFYBC-8: (**C**) Bright-field, (**D**) fluorescence, (**E**) merged images. Scale bars: (**A**,**B**) = 25 μm; (**C**–**E**) = 40 μm.

The GFP plasmid was successfully introduced into *B. pseudorignonensis* BFYBC-8 via conjugative transfer. Confocal laser scanning microscopy confirmed successful colonization of *E. crus-galli* roots by strain BFYBC-8, with bacterial cells predominantly localized in the intercellular spaces (Figure 4A,B). These observations indicate that *B. pseudorignonensis* BFYBC-8 infect and maintain its impact on *E. crus-galli* roots. Furthermore, strain BFYBC-8 exhibited preferential colonization in apical cells, root hairs, and mature zone cells at the root apex (Figure 4C–E).

### 3.5. Inhibitory Activity of Secondary Metabolites of B. pseudorignonensis BFYBC-8

**Figure 5:**
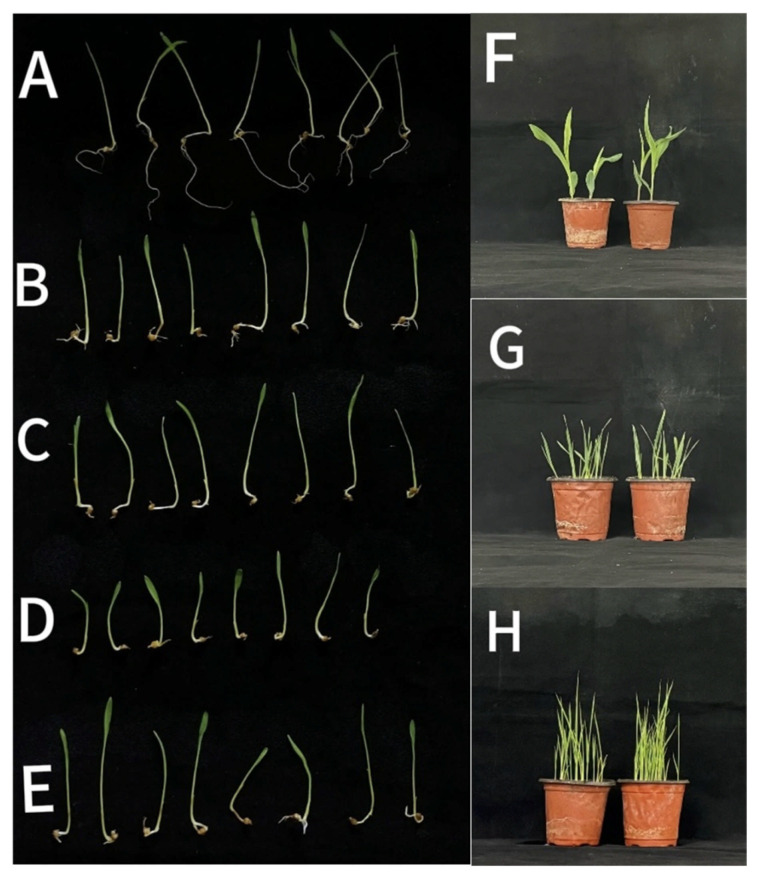
Thermal stability assay of bioactive compounds from *B. pseudorignonensis* BFYBC-8 strain, (**A**) sterile water, (**B**) 28 °C, (**C**) 60 °C, (**D**) 80 °C, and (**E**) 100 °C. The experiment on the safety of the supernatant of strain BFYBC-8 cultured at room temperature to crops, (**F**) Maize, (**G**) Wheat and (**H**) Rice.

Secondary metabolites of *B. pseudorignonensis* BFYBC-8 significantly suppressed root growth in *E. crus-galli* (Figure 5A–E). After treatment at 60 °C, 80 °C, and 100 °C, the inhibition rates of plant height were 17.1%, 20.8%, and 18.5%, respectively, while the root length were 83.8%, 84.15%, and 85.2% (Figure 5 and Table 4). However, no significant differences were observed among the various temperature treatments. These results indicate that the secondary metabolites of *B. pseudorignonensis* BFYBC-8 retained inhibitory activity against *E. crus-galli* growth under thermal stress, indicating thermal stability.

### 3.6. Crop Safety Test of B. pseudorignonensis BFYBC-8

Root irrigation with *B. pseudorignonensis* BFYBC-8 bacterial suspension resulted in no observable phytotoxicity to rice, wheat, or maize at the two-leaf stage. Based on the disease severity rating scale, all treated crop plants received a severity score of 0, exhibiting healthy growth comparable to the negative controls and showing none of the growth abnormalities (e.g., root yellowing or shortening, fragile leaves) that were characteristic of infected *E. crus-galli.* Consequently, the disease incidence was calculated as 0% for these crops. These quantitative results demonstrate that strain BFYBC-8 has no phytotoxic effects on the tested non-target crops (Figure 5F–H).

### 3.7. LC-MS Identification of Bioherbicidal Metabolites in B. pseudorignonensis BFYBC-8 Crude Extract

**Figure 6:**
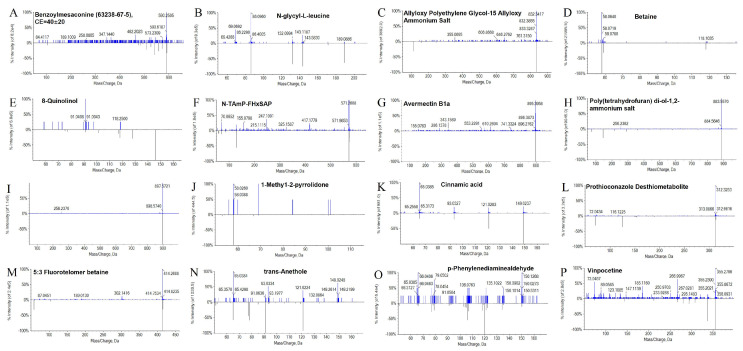
Mass spectrum of the crude extract of the herbicidal activity of strain BFYBC-8. Identified compounds: (**A**) Benzoylaconine; (**B**) N-glycyl-L-leucine; (**C**) Allyloxy Polyethylene Glycol −15 Allyloxy Ammonium Salt; (**D**) Betaine; (**E**) 8-Quinolinol; (**F**) N-TAmP-FHxSAP; (**G**) Avermectin B1a; (**H**) Poly(tetrahydrofuran) diol−1,2-ammonium salt; (**I**) Ivermectin; (**J**) 1-Methyl−2-pyrrolidone; (**K**) Cinnamic Acid; (**L**) Prothioconazole Desthiometabolite; (**M**) 5:3 Fluorotelomer Betaine; (**N**) trans-Anethole; (**O**) p-Phenylenediaminealdehyde; (**P**) Vinpocetine.

The herbicidal crude extract of *B. pseudorignonensis* BFYBC-8 was analyzed via LC-MS to identify its primary active components. The identified components spanned diverse chemical classes, including alkaloids, ethers, alcohols, ketones, and amino acids. A total of 16 compounds matched entries in the database as putative active substances produced by *B. pseudorignonensis* BFYBC-8 (Table 5 and Figure 6).

## 4. Discussion

The extensive application of synthetic herbicides in modern agriculture, while crucial for global food security, has precipitated profound ecological sustainability challenges, particularly the global escalation of herbicide resistance in weeds [1]. However, bacterial herbicides demonstrate unique advantages by reducing weed resistance development, minimizing harm to non-target species, and delivering eco-friendly impacts compared to conventional chemical alternatives [2,17]. Our current investigation identified 28 bacterial strains from non-germinating seeds exhibiting differential phyto-toxic activity against *Echinochloa crus-galli*. *B. pseudorignonensis* BFYBC-8 exhibited pronounced weed-suppressive activity through the secretion of bioactive secondary metabolites, coupled with experimentally validated crop safety. The strain exhibits no detectable phytotoxicity toward major cereal crops, including *Oryza sativa* (rice), *Triticum aestivum* (wheat), and *Zea mays* (corn). The species-specific pathogenicity of BFYBC-8 is likely mediated by differential defense mechanisms in crops versus *E. crus-galli*, particularly compositional disparities in cell wall polysaccharides and activation thresholds of the salicylic acid-mediated defense signaling pathway. This discovery epitomizes the emerging microbial herbicide paradigm by demonstrating the species-specific pathogenicity of BFYBC-8, positioning it as a dual-function biocontrol agent against *E. crus-galli* with validated crop safety.

The genus *Brucella*, long recognized as a group of zoonotic pathogens causing brucellosis in humans and livestock (e.g., cattle, sheep, and goats), has recently revealed an unexpected bioherbicidal potential through our research, a trait that remains unexplored in previous studies [27,28,29]. *Brucella pseudorignonensis* BFYBC-8 exhibited significant reductions in root length, leaf length, and overall biomass accumulation on *E. crus-galli* weeds. Moreover, *E. crus-galli* seedlings treated with the strain BFYBC-8 showed morphological abnormalities, such as weakened stems and leaves, chlorosis of foliage and stems, and overall plant distortion. *Pseudomonas fluorescens* and *X. campestris*, respectively exhibit comparable inhibitory effect on *Setaria viridis* and *Pao annua*, and these bacteria inhibit plant growth and germination by excreting extracellular metabolites [30,31]. In particular, *P. syringae* has been found effective in controlling the germination of bluegrass, wild sunflower, aster weeds, cocklebur, creeping thistle, and ragweed [32]. Previous studies have also reported the potent inhibitory activity of *Colletotrichum graminicola* B-48, *Curvularia lunata* NX1 and *Bipolaris yamadae* HXDC-1-2 against *E. crus-galli* [16,33,34]. However investigations into the bacterial biocontrol of this weed remain conspicuously limited. This discovery epitomizes the emerging bacterial herbicide paradigm through demonstrating the species-specific pathogenicity of BFYBC-8, positioning it as a dual-function biocontrol agent against *E. crus-galli* with validated crop safety.

LC-MS analysis of crude extracts revealed a diverse array of compounds produced by *B. pseudorignonensis* BFYBC-8, suggesting a multifaceted herbicidal mechanism. Cinnamic acid and trans-anethole within compounds were, respectively, identified as allelochemicals capable of disrupting weed hormonal balance and cell membrane integrity, respectively, so both of compounds may affect critical physiological processes like seed germination, root growth, and photosynthesis [35,36]. Alkaloids are ubiquitously distributed in the plant kingdom and are well-documented for their critical defensive functions, protecting plants against herbivores, pathogenic microorganisms, fungi, and occasionally competing neighboring plants [37]. Three alkaloids in the fermentation broth of strain BFYBC-8, including Benzoylaconine, Betaine, and 5:3 Fluorotelomer Betaine were identified in this study. Although no direct herbicidal activity was observed for betaine compounds itself, it is frequently employed as an adjuvant in combination with other agents to enhance herbicidal efficacy [37,38]. Notably, even after 30 min of heat treatment at 100 °C, the culture filtrate (or: supernatant) containing these secondary metabolites retained a remarkably high inhibitory effect on root length (85.2%), underscoring its exceptional thermostability. (This high thermostability suggests that the phytotoxic compounds are non-proteinaceous in nature, as most proteins denature at this temperature.). This property is highly advantageous for the potential development of a bioherbicide, as it indicates that the active ingredients would likely remain stable during storage and could withstand certain industrial processing steps that might involve heat.

It should be emphasized that this study only conducted preliminary validation of the herbicidal activity of BFYB-8 under laboratory conditions. However, field environments exhibit higher complexity, characterized by critical factors such as variations in soil physicochemical properties, climatic dynamics, and microbial community interactions, all of which may significantly impact its colonization ability and weed suppression efficacy. Consequently, subsequent research should prioritize conducting multidimensional field trials to systematically evaluate BFYB-8′s herbicidal performance stability and persistence across diverse agroecological zones. Despite these existing research gaps, the superior activity demonstrated by BFYBC-8 in biological control of *E. crus-galli* provides novel strain resources and a theoretical foundation for developing eco-friendly microbial herbicides.

## 5. Conclusions

This study establishes *B. pseudorignonensis* BFYBC-8 as a novel and promising microbial herbicide candidate, presenting a viable strategy to combat herbicide-resistant *Echinochloa crus-galli*. The strain’s efficacy, demonstrated through significant growth inhibition, is coupled with a unique mode of action involving root colonization and the production of exceptionally thermostable phytotoxic metabolites. Critically, its stringent species-specificity towards the target weed, alongside the absence of phytotoxicity in major cereals, underscores its potential for seamless integration into existing agricultural systems without compromising crop safety. The dual attributes of potent weed suppression and a favorable environmental profile position BFYBC-8 not only as a solution to address escalating resistance issues but also as a cornerstone for developing next-generation, sustainable weed management protocols.

## Figures and Tables

**Table 1:** Main experimental instrument information table.

Instrument Name	Model	Manufacturer/Source
Cryostat Microtome	CM1860UV	Leica Microsystems, Wetzlar, Germany
High-Pressure Steam Sterilizer (Autoclave)	GR60DA	ZEALWAY Instrument Co., Ltd., Xiamen, China
UV-Vis Spectrophotometer	UV-5100B	Yuanxi Instrument Co., Ltd., Shanghai China
Confocal Microscope	DMi8	Leica Microsystems, Wetzlar, Germany
Biological Microscope	BH200	Sunway Optical Technology Group Co., Ltd., Sichuan, China
pH Meter	FE28 Standard	Mettler-Toledo Instrument Co., Ltd., Shanghai, China
Pipette (Adjustable Volume Pipettor)	Research^®^ Plus	Eppendorf AG, Hamburg, Germany
Illuminated Incubator Shaker	HZ250LG	Ruihua Instrument Equipment Co., Ltd., Wuhan, China
Laboratory Water Purification System	RO D1800-E	Heqi Instrument Co., Ltd., Shanghai, China
Intelligent Illuminated Plant Growth Chamber	SPX-150-GB	Langgan Laboratory Equipment Co., Ltd., Shanghai, China
Laminar Flow Clean Bench (Double-sided)	SW-CJ-2FD	Antai Air Technology Co., Ltd., Suzhou, China
Electric Thermostatic Blow-drying Oven	DHG-9246A	Jinghong Experimental Equipment Co., Ltd., Shanghai, China
Ultrasonic Cleaner	KQ2200	Shumei Ultrasonic Instrument Co., Ltd., Kunshan, China
Digital Caliper	150T	DR. JOHANNES HEIDENHAIN Co., Ltd., Beijing, China
Refrigerated Centrifuge	5430R	Eppendorf AG, Hamburg, Germany
Flake Ice Maker	SIM-F140AY65-PC	Lingchu Environmental Protection Instrument Co., Ltd., Shanghai, China
Thermal Cycler (PCR Machine)	Mastercycler nexus	Eppendorf AG, Hamburg, Germany
Vortex Mixer	G560E	Scientific Industries, Inc., New York, NY, USA
Digital Camera	EOS R6 Mark II (EF100mm f/2.8L Macro IS USM)	Canon Co., Ltd., Beijing, China
Magnetic Stirrer	IT-09A5	Yiheng Scientific Instrument Co., Ltd., Shanghai, China
Electric Thermostatic Water Bath	DK-98-II	Tester Instrument Co., Ltd., Tianjin, China
Electrophoresis System	DYY-4C	Liuyi Instrument Factor, Beijing, China
Refrigerator (Non-frost)	BCD-218WLDPPU1	Haier Smart Home Co., Ltd., Qingdao, China
Ultra-Low Temperature Freezer	DW-86L828J	Haier Special Electrical Appliance Co., Ltd., Qingdao, China
Mini Centrifuge	D1008E	Jinggong Industrial Co., Ltd., Shanghai, China
Gel Documentation System	Gel Doc XR+	Bio-Rad Laboratories, Inc., California, CA, USA
Electronic Balance	LQ-C6002	Feiya Weighing Apparatus Co., Ltd., Shenzhen, China
Precision Electronic Balance	FA1204B	Jingke Tianmei Scientific Instrument Co., Ltd., Shanghai, China
High-Throughput Tissue Homogenizer	XU-YM-48	Xiniu Lab Instrument Co., Ltd., Shanghai, China
Induction Cooker	HK-22	Zhenyu Electric Appliance Co., Ltd., Zhongshan China

**Table 2:** Inhibitory activity of endophytic strains from non-germinated seeds against *E. crus-galli* (Preliminary screening).

Treatment	Inhibition Rate of Plant Height (%)	Inhibition Rate of Root Elongation (%)
BFYBC-1	−2.9 ± 2.3 ^kl^	−10.4 ± 13.3 ^cde^
BFYBC-2	7.9 ± 5.0 ^fghij^	6.5 ± 4.8 ^bc^
BFYBC-3	−3.4 ± 4.1 ^kl^	0.3 ± 9.4 ^bcd^
BFYBC-4	12.4 ± 2.7 ^def^	4.7 ± 3.9 ^bcd^
BFYBC-5	9.0 ± 2.3 ^fhgij^	4.8 ± 7.3 ^bcd^
BFYBC-6	9.9 ± 2.4 ^fghi^	4.0 ± 6.3 ^bcd^
BFYBC-7	2.2 ± 1.0 ^jk^	8.6 ± 6.4 ^b^
BFYBC-8	25.1 ± 1.8 ^ab^	86.3 ± 2.0 ^a^
BFYBC-9	10.3 ± 7.9 ^fgh^	−2.1 ± 5.9 ^bcd^
BFYBC-10	11.8 ± 1.9 ^efg^	−0.5 ± 8.2 ^bcd^
BFYBC-11	25.7 ± 2.5 ^a^	11.3 ± 4.5 ^b^
BFYBC-12	30.3 ± 1.7 ^a^	0.0 ± 11.1 ^bcd^
BFYBC-13	14.0 ± 2.9 ^def^	−0.2 ± 4.3 ^bcd^
BFYBC-14	18.8 ± 2.5 ^bcd^	10.3 ± 8.5 ^b^
BFYBC-15	3.4 ± 1.4 ^ijk^	−2.8 ± 6.9 ^bcde^
BFYBC-16	4.6 ± 4.8 ^hij^	2.4 ± 4.4 ^bcd^
BFYBC-17	24.4 ± 2.1 ^abc^	−2.1 ± 6.9 ^bcd^
BFYBC-18	17.7 ± 4.7 ^cde^	4.2 ± 17.0 ^bcd^
BFYBC-19	−4.8 ± 2.1 ^l^	−19.2 ± 6.9 ^e^
BFYBC-20	9.1 ± 2.7 ^fghi^	−1.6 ± 9.5 ^bcd^
BFYBC-21	5.3 ± 2.6 ^ghij^	−9.4 ± 8.7 ^cde^
BFYBC-22	7.7 ± 3.1 ^fghij^	−10.8 ± 10.3 ^de^
BFYBC-23	8.3 ± 2.1 ^fghij^	3.4 ± 6.0 ^bcd^
BFYBC-24	8.8 ± 4.6 ^fghij^	5.4 ± 5.7 ^bcd^
BFYBC-25	12.1 ± 3.2 ^defg^	−2.4 ± 7.2 ^bcde^
BFYBC-26	7.3 ± 3.7 ^fghij^	−2.0 ± 10.8 ^bcd^
BFYBC-27	9.1 ± 3.5 ^fghi^	1.9 ± 2.2 ^bcd^
BFYBC-28	4.3 ± 2.6 ^hij^	−4.7 ± 11.9 ^bcde^

Note: The values in the Table are the average value ± the standard deviation (*n* = 3), and the different letters within the same column indicate that the differences are significant at the 0.05 level. The same below.

**Table 3:** Inhibitory activity of endophytic strains from non-germinated *E. crus-galli* seeds against *E. crus-galli* (Re-screening).

Treatment	Inhibition Rate of Plant Height (%)	Inhibition Rate of Root Elongation (%)
BFYBC-8	16.7 ± 1.8 ^a^	85.1 ± 3.7 ^a^
BFYBC-11	1.7 ± 3.3 ^b^	2.3 ± 6.6 ^b^
BFYBC-12	−1.4 ± 4.7 ^b^	1.9 ± 6.3 ^b^
BFYBC-17	2.8 ± 3.2 ^b^	5.2 ± 6.4 ^b^

Note: The values in the Table are the average value ± the standard deviation (*n* = 8), and the different letters within the same column indicate that the differences are significant at the 0.05 level.

**Table 4:** Thermal stability test results of pathogenic active substances of *B. pseudorignonensis* BFYBC-8.

Temperature	Inhibition Rate of Plant Height (%)	Inhibition Rate of Root Elongation (%)
28 °C	17.0 ± 2.4	83.0 ± 1.3
60 °C	17.1 ± 0.4	83.8 ± 1.6
80 °C	20.8 ± 2.9	84.1 ± 2.5
100 °C	18.5 ± 1.4	85.2 ± 0.7

Note: The values in the Table are the average value ± the standard deviation (*n* = 8).

**Table 5:** LC-MS analysis results of crude extract of herbicidal activity of *B. pseudorignonensis* BFYBC-8.

Material Type	Molecular Formula	Ion Binding Form	Mass/Charge	Retention Time
Benzoylaconine	C_24_H_29_NO_5_	[M + H]+	590.2509	1.27
N-glycyl-L-leucine	C_8_H_16_N_2_O_3_	[M + H]+	189.0862	1.36
Allyloxy Polyethylene Glycol-15	C_4_H_5_O-(C_2_H_4_O)_15_-C_4_H_5_O·NH_4_	[M + Na]+	832.3412	1.99
Allyloxy Ammonium Salt				
Betaine	C_5_H_11_NO_2_	[M + H]+	118.0642	2.18
8-Quinolinol	C_9_H_7_NO	[M + H]+	146.059	2.18
N-TAmP-FHxSAP	C_15_H_21_F_13_NO_5_S	[M + H]+	571.2601	2.62
Avermectin B1a	C_48_H_72_O_14_	[M + H]+	895.3938	3.95
Poly(tetrahydrofuran) diol-1,2-ammonium salt	C_48_H_96_O_12_·NH_4_	[M + H]+	883.5565	6.78
Ivermectin	C_48_H_74_O_14_	[M + H]+	897.5727	6.79
1-Methyl-2-pyrrolidone	C_5_H_9_NO	[M + H]+	100.1111	8.95
Cinnamic Acid	C_9_H_8_O_2_	[M + H]+	149.0227	9.04
Prothioconazole Desthiometabolite	C_14_H_15_F_3_N_2_O_2_S	[M + H]+	312.3251	11.76
5:3 Fluorotelomer Betaine	C_12_H_16_F_9_NO_3_S	[M + H]+	414.2678	12.08
trans-Anethole	C_10_H_12_O	[M + H]+	149.0229	20.95
p-Phenylenediaminealdehyde	C_9_H_11_NO	[M + H]+	150.0255	21.58
Vinpocetine	C_21_H_26_N_2_O_3_	[M + H]+	355.0682	25.74

## Data Availability

The original contributions presented in this study are included in the article. Further inquiries can be directed to the corresponding author.
